# Development of cancer policies between Europe Against Cancer Programme and Europe’s Beating Cancer

**DOI:** 10.1093/eurpub/ckac131.310

**Published:** 2022-10-25

**Authors:** T Albreht, M Jelenc

**Affiliations:** 1 Centre for Health Care, National Institute of Public Health, Ljubljana, Slovenia; 2 Department of Public Health, Faculty of Medicine, University of Ljubljana, Ljubljana, Slovenia

## Abstract

**Issue/problem:**

European Union has legally certain limitations in developing joint policies for services, activities and interventions that belong to the national competencies. Cancer control and cancer policy occupy a special place as they have received special attention throughout the last 40 years.

**Description of the problem:**

European Union launched its first important disease policy with the start of the Europe Against Cancer Programme in 1984/85. It was a step, supported by a few interventions and activities, which facilitated the development of several important policy tools. After its closure in 2003, there was some unease, especially in view of the forthcoming biggest enlargement of the EU in 2004. Several activities revived the EU cancer policy and these processes culminated in the adoption of the Europe’s Beating Cancer Plan. There were some doubts whether the latter would be feasible but a careful development process at different levels resulted in success.

**Results:**

A policy analysis will be presented that will introduce key steps and documents that mark the period between the two milestones - Europe Against Cancer Programme (EACP) and Europe’s Beating Cancer Plan (EBCP). We will present the development of various tools as well as the important outcomes of the different policies and interventions triggered by the two documents. The period between 2003 and 2019 will be elaborated in view of the adoption of EBCP. Three Joint Actions and their various outputs with a strong impact on the cancer policy development, contributing also to the development of flagships and actions of EBCP will be presented as well.

**Lessons:**

EU top level decisions and policies can be developed harmoniously and to the benefit of both - the EU and the Member States - when launched in a joint process and with the involvement of all the relevant stakeholders.

**Key messages:**

• EU policy documents are important drivers for the development of health policy tools at both the EU level as well as at the Member State level.

• A combination of top-level documents with carefully developed policy tools enables the development of cancer policies acting at both EU and Member State level.

